# Automated Sample Preparation Platform for Mass Spectrometry-Based Plasma Proteomics and Biomarker Discovery

**DOI:** 10.3390/biology3010205

**Published:** 2014-03-11

**Authors:** Vilém Guryča, Daniel Roeder, Paolo Piraino, Jens Lamerz, Axel Ducret, Hanno Langen, Paul Cutler

**Affiliations:** F. Hoffmann-La Roche Ltd., Pharma Research and Early Development (pRED), Translational Technologies and Bioinformatics, 124 Grenzacherstrasse, Bldg. 93/4.38, 4070 Basel, Switzerland; E-Mails: vilem.guryca@roche.com (V.G.); daniel.roeder@roche.com (D.R.); ppiraino@gmail.com (P.P.); jens.lamerz@roche.com (J.L.); axel.ducret@roche.com (A.D.); hanno.langen@roche.com (H.L.)

**Keywords:** automation, mass spectrometry, plasma, proteomics

## Abstract

The identification of novel biomarkers from human plasma remains a critical need in order to develop and monitor drug therapies for nearly all disease areas. The discovery of novel plasma biomarkers is, however, significantly hampered by the complexity and dynamic range of proteins within plasma, as well as the inherent variability in composition from patient to patient. In addition, it is widely accepted that most soluble plasma biomarkers for diseases such as cancer will be represented by tissue leakage products, circulating in plasma at low levels. It is therefore necessary to find approaches with the prerequisite level of sensitivity in such a complex biological matrix. Strategies for fractionating the plasma proteome have been suggested, but improvements in sensitivity are often negated by the resultant process variability. Here we describe an approach using multidimensional chromatography and on-line protein derivatization, which allows for higher sensitivity, whilst minimizing the process variability. In order to evaluate this automated process fully, we demonstrate three levels of processing and compare sensitivity, throughput and reproducibility. We demonstrate that high sensitivity analysis of the human plasma proteome is possible down to the low ng/mL or even high pg/mL level with a high degree of technical reproducibility.

## 1. Introduction

Mainly due to their sensitivity and multiplexing capabilities, mass spectrometry (MS) based proteomics approaches have emerged as one of the indispensable tools for biomarker discovery in clinical research [1]. Despite limited proteome coverage, plasma proteomics by mass spectrometry remains one of the few available methods to systematically characterize molecular alterations at the protein level in an unbiased way [2]. It has long been recognized that sample preparation and fractionation is key to successful profiling of the plasma proteome. In general, the common MS sample preparation pipeline consists of sample processing (protein extraction, denaturation, derivatization and clean-up), followed by protein digestion via trypsin, and detection of released peptides in a mass spectrometer. The delivery of the peptides to the mass spectrometer is often coupled to a nanoflow chromatography system. Despite the increased sensitivity concomitant with nanoflow chromatography the ability to detect low abundant proteins in plasma is still challenging. To increase the sensitivity of the analysis further, various fractionation techniques can be employed, either at the peptide or the protein level. Some traditional protein fractionation methods, such as gel electrophoresis have been employed but have finite reproducibility, caused, in particular, by manual handling of samples [3]. Even after the implementation of advanced automation/robotics the pipeline remains rather complex and is not generally practicable for all types of clinically relevant samples.

Sensitivity of analysis therefore remains a significant challenge for human plasma (or serum), where protein expression occurs over a very large dynamic range (~10^12^) and the proteins associated with tissue damage and cellular response to disease and drugs are low in comparison to the constitutive plasma proteins such as albumin and immunoglobulins. The need to increase the sensitivity of the plasma proteome by reproducible fractionation and more sensitive detection has long been realized [4,5]. It has been estimated, that the thirty most abundant plasma proteins constitute approximately 99% of the protein mass of human plasma [6]. As the dynamic range of analysis by mass spectrometry is finite and effectively scaled to the most abundant peptides, effective removal of the most abundant proteins can have a significant effect on the sensitivity achieved. Removal of individual proteins has been demonstrated and commercially available columns exist for removal of these proteins by immunodepletion, e.g., MARS (Agilent) or Seppro (Sigma). These columns tend to remove between 10 and 14 of the most abundant proteins by multiple immune-depletion. Qian *et al.*, have used a similar immunoaffinity system based on chicken IgY [7]. Others have used alternative methods for protein fractionation. These include strong cation exchange and RP HPLC of the intact proteins prior to digestion, These often reflect enrichment for subsequent targeted analysis such as selected reaction monitoring [8,9,10,11,12], where enrichment of only a limited number of proteins is required. Whiteaker achieved low ng/mL sensitivity using immunocapture of selected proteins [13].

Pre-fractionation using single dimension chromatography alone is not always adequate in order to improve sensitivity sufficiently to reproducibly detect very low abundant proteins. The utilization of complex multidimensional fractionation schemes, e.g., combining depletion and fractionation, may resolve the sensitivity issue. However, such multiple-step fractionation routinely introduces process variability, which prevents the precision and accuracy of quantitation as well limits analytical throughput. It is therefore necessary to consider the automation of the process to reduce cycle time, manual handling and technical variability.

Zolotarjova *et al.*, demonstrated depletion on the multiple affinity removal system (Human-14, Agilent), followed by fractionation of flow-through proteins on reversed-phase column (mRP-C18, Agilent) [14]. Shen *et al.*, introduced the concept of protein digestion performed directly after the depletion, *i.e.*, without fractionation at the intact protein level [15]. The resulting peptide mixture was submitted for exhaustive multidimensional LC separations (“MudPit”) [16]. Immunoaffinity depletion with the ProteomeLab™ (IgY-12, Sigma) has also been demonstrated. Second and third dimension separations of the enriched proteome were performed on the platform utilizing 2D isoelectric focusing and RP-HPLC. Jones *et al.*, recently reported a comparison of plasma proteomics following immunodepletion comparing a range of state of the art mass spectrometers and suggest some potential future improvements in both LC and MS performance [17]. 

Jmeian *et al.*, have described a novel LC system enabling capture of proteins from the depletion via a reversed phase material optimized for injections of *circa* 25 µL plasma [18]. In a subsequent paper he further refined the methodology, employing tandem affinity columns for the depletion and fractionation of low-abundance plasma proteins by tandem immobilized metal-ion affinity chromatography columns and capture on reversed phase (RP) column [19]. Pan *et al*. reported a combination of pre-digestion RP chromatography and post-digestion cation exchange to characterize the human plasma proteome in patients with pancreatic cancer and chronic pancreatitis [20]. An alternative automated setup, that combines an immuno-depletion step, protein capture on trap and subsequent RP protein fractionation, is described by Cellar *et al.*, [21]. The chromatography system was successfully scaled to deplete/fractionate *circa* 40 µL plasma. A limitation however was that this methodology is not compatible with on trap derivatization of proteins. In addition, the material of the trap, a silica particle based stationary phase, is not generally compatible with harsh derivatization solvents. One option to overcome this caveat is an on-line multidimensional system, consisting of a pH gradient on strong anion exchange chromatography of native proteins in the first dimension, subsequent trapping and on-column reduction/alkylation on C4 trap columns and reverse phase separation of the alkylated proteins in the second dimension, followed by on-column tryptic digestion and electrospray MS detection. In the future, advanced mass spectrometric techniques, such as parallel reaction monitoring (PRM) and SWATH, offer real promise for increased plasma proteomic analysis [22,23,24].

Inspired by recent papers, we have developed and evaluated a generic sample preparation platform, targeting critical biological samples (e.g., plasma, serum, urine, CSF), which works at a micro-scale (e.g., approximately 10 µL plasma) and is sufficiently sensitive to detect protein levels significantly below 1 µg/mL. The system combines all the features of automated depletion and fractionation but in addition, it makes performing protein derivatization (e.g., reduction of cysteines, alkylation, isotope labeling) and subsequent clean-up feasible, directly in the automated instrument. Modified and cleaned-up proteins can therefore be used for the digestion and nano-LC/MS, without any manual post-cleaning steps, such as cartridges filled with reversed phase material. The feasibility of such chemical derivatization of proteins immobilized on solid hydrophobic support has been tested in several manual protocols [25,26]. In the method we describe here, we extend the automation and develop the concept of multidimensional separation and on line processing for the analysis of a complex a biofluid such as human plasma.

In developing this methodology it was critical that we maintained the ability to perform quantitative analysis. As such we sought to establish the robustness not only of the overall process but of each additional step in the protocol. This has enabled us to identify, and in some cases improve, the key stages of the process. In addition we have been able draw a comparison between the analyses of neat plasma, immunoaffinity depleted plasma and plasma which has undergone both immunodepletion and subsequent protein fractionation by RP HPLC. Critically for each of these processes we can identify the effect not only in terms of protein sensitivity but also in terms of process variability (as measured by the coefficient of variation, *etc*.) and on process throughput. At this stage we have tested the platform as a multiplexed label-free protein assay, targeting a maximal number of identifications per sample and bringing knowledge of their reproducibility values. Nevertheless, the system can be in principle easily employed as a generic platform for bio-fluid sample preparation in targeted MS detection strategies, such as selected reaction monitoring (SRM) or even for immunoassays [27].

## 2. Materials and Methods

EDTA-stabilized human plasma was obtained from Bioreclamation LLC (USA). Trypsin Gold was obtained from Promega (Switzerland). All other reagents were purchased at the highest analytical grade (if not otherwise indicated) either from Sigma-Aldrich, Fluka, Merck, Thermo Scientific, or Pierce.

### 2.1. Automated Multi-Dimensional Chromatography

**Figure 1:**
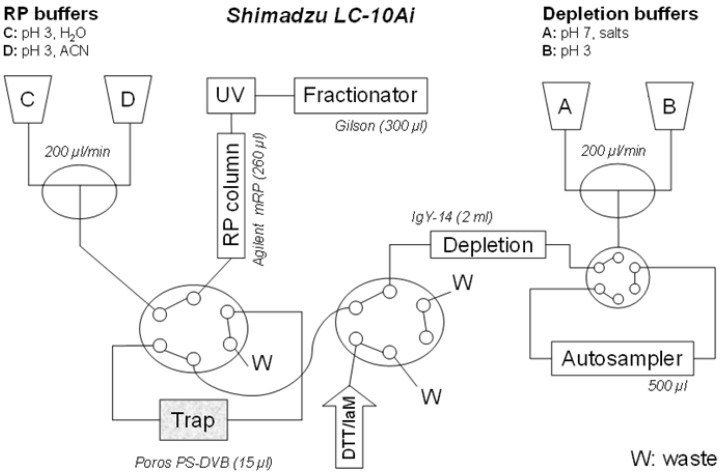
An automated setup for the multidimensional LC system, allowing an on-line integration of depletion, protein derivatization and fractionation. Connection of depletion and RP columns into a single separation unit, through common LC pumps (**A**, **B**, **C**, **D**), capillary linkages and two 6-port valves (X, Y). Pumps **A** and **B** are delivering buffers for loading the proteins on-trap, and, if deployed, for a depletion procedure. The pumps **C** and **D** provide for acetonitrile gradient in RP protein fractionation. The reagents for derivatization (dithiothreitol, iodoacetamide) and desalting (water) are injected through the valve Y.

The proposed configuration of the automated liquid chromatography system for on line multi-dimensional chromatography and sample reaction is shown in Figure 1. The configuration utilizes four LC-10 Ai liquid chromatography pumps, autosampler SIL-20 AC, diode array detector SPD-M20 A, communications bus module CBM-20A two CTO-20 AC column ovens. Two Rheodyne 9710-050 six-port valves (all purchased from Shimadzu Reinach, Switzerland). As an autosampler, Valves Gilson 215 liquid handler Nebula series (USA) was connected through a 0.25 mm ID PEEK linkage (VICI, USA).

For depletion column, a Seppro IgY-14 LC-2 (Sigma), customized for depletion of *circa* 10 µL plasma was used with vendor provided buffers. Flow-through was collected on polymeric trap from monolithic poly(styrene-divinylbenzene) with a patented bimodal pore size distribution for rapid mass transport: Poros R1 5 × 2 mm, Dr. Maisch HPLC, Morvay Analytik, Basel, Switzerland. After proteins are immobilized on the trap, derivatization solvents (dithiothreitol followed by iodoacetamide) are injected manually through syringes. The RP chromatography was performed and proteins are eluted by acetonitrile gradient onto Agilent MRP-C18, 2.1 × 75 mm and 10 fractions collected. The Poros material (polystyrene/divinylbenzene)—was selected as it is inert to harsh pH, solvent, chemical, temperature changes and pressure drops. It also demonstrates the excellent permeability needed when connected in-line with IgY depletion column. For efficient separation of intact proteins the Agilent C-18 demonstrates retention power higher than polystyrene in the trap column (equivalent roughly to C4). This allowed an efficient refocusing effect separation power.

10 µL plasma is diluted to 500 µL with mobile phase (IgY buffer) and loaded onto the depletion column (IgY-14) at 200 µL/min (25 °C), The flow-through is collected on the trap POROS column for 25 min (55 °C). The depletion column is then washed and equilibrated with the IgY elution buffer (1500 µL/min) using a triangle gradient of 0%–100% (5 min) and 100%–0% (15 min), followed by equilibration for 15 min. The total cycle time is 60 min.

After the proteins are immobilized on the trap derivatization is performed using (25 mM dithiothreitol followed by 100 mM iodoacetamide injected manually via syringes (50 µL/min for 2 min), and incubated for 10 min each (55 °C). After desalting the RP chromatography was performed and proteins are eluted by the following acetonitrile gradient (200 µL /min, 55 °C, pH3): 0% for 3 min, 0%–30% (1 min), 30%–60% (15 min), 60%–90% (0.1 min), 90%–95% (5 min), 95% (10 min), 95%–0% (0.1 min) and 0% (10 min). Separation column Agilent MRP-C18 (2.1 × 75 mm) was used and 10 fractions collected, within the total cycle time of 45 min. Using this protocol operating pressures should not exceed 25 bar. The efficiency of the automated derivatization protocol was compared to the manual method. The LC-MS analyses of digests revealed the number of alkylated peptides was equal and with lower level of miss-cleavages [28]. 

### 2.2. Standard Plasma Preparations

To benchmark the automated platform in terms of sensitivity, two additional non-automated sample preparations were performed. The first analysis used 1 µL plasma using standard protocol as described above. In a second approach, further fractionation was achieved by depleting the SepproIgY-14 depleted material using a Seppro SuperMix column, depleting approximately 99% of the plasma protein mass. This material was subsequently fractionated by RP separation in to 20 fractions.

### 2.3. Nano-Flow Liquid Chromatography and Mass Spectrometry

The LC-MS measurements were performed on a LTQ-Orbitrap (Thermo Electron, Bremen, Germany), coupled to an Ultimate 3000 nano-flow chromatographer (LC-Packings, Amsterdam, The Netherlands). Peptides were separated on an in-house packed PicoTip nano-column (FS360-75-8-N-5-C20, New Objective, USA) using Reprosil-Pur C18-AQ (Dr. Maisch, Germany, particle size 3 µm). reversed phase material of 75 µm ID and the length of 15 cm.

The samples were loaded (5 µL) directly on-column at the constant pressure of 220 bar with the mobile phase A. Mobile phases consisted of (A) 0.5% acetic acid, 97.5% water and 2% acetonitrile (v/v/v) and (B) 0.5% acetic acid, 20% water in 79.5% acetonitrile (v/v/v). Following the 13 min long loading step, the flow-rate was altered to 0.250 µL/min and the linear gradient started (0%–44% B in 60 min). The column was washed with 100% B for 15 min and re-equilibrated with 0% B for at least 25 min. After each five runs, technical replicates of standard samples were added, in order to check the consistency of stability MS-signal and reproducibility of peptide retention behavior. As a standard mixture, 50 fmol loads of six proteins lyophilized digests were utilized (LC Packings, Amsterdam, The Netherlands).

Peptide ions were electrosprayed into the mass spectrometer at constant voltage +2 kV. Before any batch of analyses, the spectrometer was tuned to singly charged 1222 Da calibration peak of Ultramark standard calibration mixture (Thermo Electron, Bremen, Germany). During the acquisitions, only doubly and triply charged ions were selected (mass/charge range 400–1650, full scan resolution set at 30,000 at m/z 400). The auto-lock mass accuracy option was switched on (445.12003 Da). In the scanning sequence, every single MS scan was followed with five data dependent MS/MS acquisitions of the most abundant ions from the survey scan (CID, normalized collision energy 35%). The tandem mass scanning was performed exclusively in the LTQ cell. Dynamic exclusion was applied for 30 s (single repeat count).

### 2.4. Data Processing and Analysis

Data were processed through Sequest search engine V.27. The search was performed against the SwissProt database (taxonomy: human) containing 32,603 proteins (including spliced variants) concatenated with its decoy to enable false discovery rate calculation. The search parameters were set to: peptide tolerance: 5 ppm (MS), fragment mass error 0.5 Da (MS/MS), with fixed (methionine oxidation) and variable (cysteine carboxyamidomethylation) modifications. Peptides must follow a strict trypsin cleavage pattern with only one missed cleavage allowed.

An in-house built protein scoring algorithm (“Clotho”) was used to determine the protein false discovery rates for each database search using an input spectrum False Discovery Rate of 2.5% as minimum threshold. Proteins identified as single peptide hits were allowed and the maximum protein FDR was estimated to ~3%–4%, with more than 98% of the proteins displaying protein scores better than 1% False Discovery Rate. The peptide mapping was done in Genedata Refiner MS software. 

The mean and standard deviation of each peak were calculated using R-software and the coefficient of variation was derived and used as estimate of repeatability and reproducibility. Reproducibility refers to three replicates obtained from different columns for method “undepleted”, and to ten replicates obtained from different operating conditions for method “depleted” (conditions were confounded in the 2^k^ factorial design, including two sample preparations, two different depletion columns, *etc*. Reproducibility of each of the ten fractions for method “fractionated” refers to four replicates obtained from two columns and two TRs. Repeatability refers to five consecutive runs under the same conditions for a sample arbitrary selected from set depleted. Both measures of precisions were assessed on the raw data and on normalized data (quantile normalization, with peptide intensities constrained to comparable distributions across samples and methods).

## 3. Results and Discussion

In order to establish a new methodology for biomarker discovery in human plasma, we have created a novel semi-automated multidimensional chromatography process, combining most of the manual steps used in common proteomics protocols. The protocol allows for both immunodepletion of the 14 most abundant proteins starting from as little as 10 µL plasma (nominally 600 µg protein equivalent in whole plasma). The 14 proteins removed are serum albumin, IgG, fibrinogen, transferrin, IgA, IgM, haptoglobin, alpha2-macroglubulin, alpha-1-acid glycoprotein, alpha-1-antitrypsin, apolipoprotein A1, apolipoprotein A2, complement C3 and apolipoprotein B. Similar to other reports of such plasma immunodepletion, this aspect of the process removes approximately 90% of the total proteins mass The proteins of interest elute in the unbound fraction and therefore the flow through can be directly processed using the valve system shown in Figure 1. They are eluted directly onto a trap column, where on line reduction and alkylation can be performed. The on line reduction and alkylation is critical for reproducible protein chromatography and subsequent trypsin digestion.

The reduced and alkylated proteins (60 µg) are subsequently separated on-line via a RP HPLC column. Typically 10 to 20 fractions, each of 300 µL, are collected and the 3–10 µg of fractionated protein is digested by trypsin. Currently the process has not been developed for on-line digestion and this occurs off-line. Following digestion the individual fractions are subsequently analyzed by LC-MS/MS and raw data were processed by Sequest. The protein False Discovery Rate was calculated and the extracted ion count profiles of identified peptides were determined.

Critically, the design of the separation is modular enabling processing of whole human plasma, inclusion of immunodepletion to remove 90% of the protein mass and the subsequent option for separation of the immunodepleted material by RP HPLC. This permits the choice of three levels of fractionation with the concomitant levels of sensitivity. In the current study the effect of the three levels of fractionation on the key assay performance criteria of sensitivity, reproducibility and throughput were established as shown in Table 1. To establish the direct effect of automation undepleted plasma was processed in an offline and in an automated fashion. It is notable that whilst manual analysis was possible with 1 µL whole plasma, the current automated system required 10 µL. This reflects the minimum sample requirement for reproducible automation.

**Table 1:** A summary of the performance of the automated analysis of human plasma at three sensitivity levels. The key parameters for samples volume requirements and performance criteria of number of unique proteins identified, the estimated low limit of detection in µg, ng or pg of protein per mL plasma, the median coefficient of variation, and time for completion of a nominal 100 sample study. The performance for whole plasma is given for both the automated and manual process. The performance for the plasma post immunodepletion and RP HPLC fractions is given for both a 10 and 20 fraction collection. Note that the detailed statistical analysis of reproducibility and therefore the coefficient of variation (CV) estimation was not made for the 20 fraction RP HPLC separation. See text for further explanation.

Workflow	Undepleted	Undepleted	Depleted	Depleted with 10 RP Fractions	Depleted with 20 RP Fractions
Sample volume (µL)	1	10	10	10	250
Automation	No	Yes	Yes	Yes	No
Identification power (proteins) *	80–100	80–100	100–150	300–500	600–800
Sensitivity limit	50 μg/mL	50 μg/mL	1 μg/mL	1 ng/mL	100 pg/mL
Reproducibility (median CV)	30%	12%	21%	26%	ND
Technical replicates of plasma sample preparations/Total LC-MS runs, incl. repeated measurements	7/21	16/19	16/16	4/40	2/40
Throughput (100 samples)	10 days	10 days	10 days	100 days	200 days

In addition, in order to calculate the three key parameters sensitivity, reproducibility and throughput the automated mode was run with all three levels of fractionation. For assessment of reproducibility as defined in Table 1 by the mean % CV the RP HPLC fractionation was run with 10 fractions collected. A subsequent analysis collected 20 fractions and this is also reported in Table 1 to establish sensitivity and throughput. The use of 20 fractions was not tested for reproducibility at this stage due to the complexity of the statistical testing; however this could be tested at a later date.

Whether in automated mode or not the analysis of whole undepleted plasma enabled the detection of 80–100 proteins. The final detection limit depending on the protein FDR identification rate applied (1%–2%, incl. single hits). Common plasma proteins were in the range >50 µg/mL as determined by other means (Table S1). As expected, at this lowest sensitivity operation the proteins identified included commonly observed and relatively highly abundant plasma proteins such as albumin, ceruloplasmin, vitamin D binding protein and fetuin A. These are proteins noted by many other studies which have analyzed whole plasma. Of note is that the calculated mean CV for automation was around 12% compared to 30% for the manual process. Although the volume of plasma was different it does appear that the automation process significantly reduces the technical CV observed during analysis.

**Figure 2:**
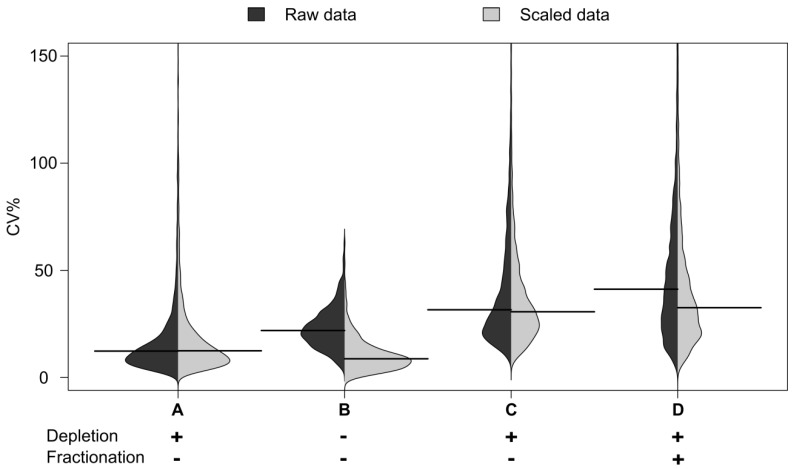
Distribution of CVs by sample group. Distribution of CV% for peak areas and their medians from peptide maps. Automated workflows: **B** (no depletion), **C** (95% depletion) and **D** (95% depletion and fractionation, fraction where the peptide is most abundant is shown). **A**: Benchmarking with an arbitrary plasma sample representing precision of the LC-MS measurement itself.

Following immunodepletion of the 14 most abundant proteins, the sensitivity was seen to increase, with 150–200 proteins identified with a nominal detection limit (based on other reports of the concentration of proteins observed) of approximately 1 µg/mL (Table S1). Proteins observed in the depleted fraction not previously seen in whole plasma included von Willebrand factor, C-reactive protein and serum paraoxonase 3. Reproducibility for each workflow is defined as the median of the distribution of CVs% across scaled data (Figure 2). The median CV for the immunodepleted material was seen to be 21%, This is higher than the 12% CV seen for automated whole plasma analysis, reflecting the increased processing. However it was encouraging that the median CV was still below the 30% seen for whole plasma on the manual process. The automation therefore appears to enable increased fractionation without dramatic loss of technical reproducibility.

The sensitivity limit of each method was determined by cross-referencing protein identifications with the circulating concentration of those proteins in plasma as reported in a previous report from our laboratory [29].

Following immunodepletion and further fractionation into 10 fractions by RP HPLC the number of protein rose further to 300–500 and the nominal lower limit of detection was at the 1 ng/mL. The level is that expected for tissue leakage products in plasma and the level believed to be approaching that necessary for effective biomarker discovery. This was reflected in the nature of the proteins observed. Proteins seen after depletion and fractionation by RP HPLC not previously seen, included human beta-1,2-*N*-acetylglucosaminyltransferase I gene (MGAT1), which controls complex and hybrid *N*-glycan synthesis, WNT1 inducible signaling pathway protein 2 (WISP2) and Desmoglein 3 a calcium-binding transmembrane glycoprotein (DSG3). The mean technical CV was 26% for this process. Therefore, despite the extra processing and the high level of fractionation, the CVs still appear lower than for whole plasma using the manual process, illustrating the benefit of the automation to retain technical reproducibility. When the number of fractions on the RP HPLC was increased from 10 to 20 the nominal sensitivity is further improved by approximately another order of magnitude (*circa* 1 pg/mL).

The distribution of CVs by sample group from the peptide maps is shown in Figure 2. The distribution of the CVs for the undepleted plasma is shown in B: The distribution for the automated workflows with 95% depletion in C and that for depletion and fractionation in D.

A caveat to the increase in sensitivity without major loss of reproducibility is the throughput in the most sensitive mode. The RP HPLC by its very nature creates 10–20 fractions from each sample and this has a multiplicative effect on the running time as shown in Table 1. As a model we assume an appropriately powered clinical study would require *circa* 100 plasma samples. Whole or depleted plasma would be processed in 10 days which is considered efficient, however the RP HPLC fractionation, based on 10 fractions would require 100 days and 20 fractions as much as 200 working days to complete analysis. It may be possible to select fractions for analysis to limit timing, however this does limit the whole proteome analysis.

Nevertheless, using the 14 protein depletion (Seppro IgY-14 LC-2) followed by reverse phase fractionation in to 10 fractions it was possible to increase the sensitivity limit from 50 µg/mL to 1 ng/mL in plasma without dramatic loss of reproducibility. There is also clearly further scope for further improvements in performance. A noted limitation of the current process is that since the samples are fractionated in to 10 or 20 fractions by HPLC the throughput drops dramatically, reflecting the increased number of analyses.

Table 2 is representative of the coverage obtained for two proteins, a relatively abundant plasma protein human apolipoprotein E (ApoE) which has been recorded in the literature at *circa* 30 µg/mL and a relatively low abundant protein insulin like growth factor 2 (IGF2) which is recorded at approximately an order of magnitude lower. For these two proteins the peptide count was recorded and is clearly higher for ApoE than IGF2 in the respective samples. This is also reflected in the peak area recorded for a representative peptide sequence from each protein. Interestingly despite the low abundance the relative CV for IGF2, whilst higher than for ApoE, is not dramatically so and within an acceptable range (e.g., <30%).

**Table 2:** Examples of a high and a low abundance protein from plasma. Apolipoprotein E (ApoE) is typically reported at about low µg/mL (*circa* 30–40 µg/mL) in the literature and insulin like growth factor 2 (IGF2) at the low ng/mL (*circa* 10–20 ng/mL). As shown each was detected, however ApoE was identified by 10, 12 and 24 unique peptides in the undepleted, depleted and depleted plus reverse phase respectively; whereas IGF2 was identified by 0, 3 and 6 peptides respectively.

	Human ApoE			Human IGF2		
Workflow	Undepleted	Depleted (95%)	Depleted (95%) and RPHPLC (10 fractions)	Undepleted	Depleted (95%)	Depleted (95%) and RPHPLC (10 fractions)
Peptide Count	-	4	71	-	1	11
Sequence	QWAGLVEK			SCDLALLETYCATPAK		
Peak Area	-	4.2 × 10^6^	2.7 × 10^7^	-	2.9 × 10^5^	21 × 10^8^
Mean CV%	-	12.7	9.7	-	21.4	11.7

**Figure 3:**
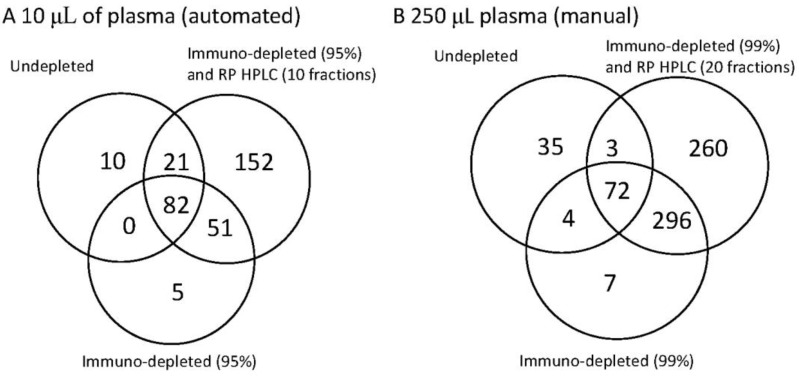
The identification power of three workflows on human plasma illustrated as a Venn diagram to demonstrate the overlap between the methods. Each Venn diagram shows the number of unique proteins detected for undepleted whole plasma, plasma depleted by immunoaffinity and plasma depleted by immunoaffinity and subsequent reverse phase separation. In diagram (**A**) (on the left) 10 µL whole plasma was analysed. Where immunodepletion was performed the most abundant 14 proteins were removed and where RP HPLC was performed 10 fractions were collected. In diagram (**B**) (on the right) the starting material was increased to 250 µL plasma. This then enabled higher fractionation. Where immunodepletion was performed the most abundant 14 proteins were removed and a further immunodepletion (Supermix) was used to remove up to 99% of the total protein mass. Where RP HPLC was used 20 fractions were collected. The left hand panel (**A**) therefore represents the options for the current automation, whereas (**B**) represents the potential for the extreme of fractionation.

In order to show the future potential for increased sensitivity we compared automated analysis of 10 µL plasma at the three levels of fractionation with the manual processing of 250 µL plasma at the at three levels of fractionation (Figure 3). It has been noted that further increases in sensitivity may be achieved by using a Supermix column, which immunodepletes 99% of protein mass representing approximately 100 of the highest abundant plasma proteins as [7].

The 10 µL plasma was isolated, as before, with the immunodepletion of the most abundant 14 proteins and the RP HPLC, where performed, was run to collect 10 fractions. For the manual high sensitive mode the immunodepletion was performed first to remove the most abundant 14 proteins, then with the Supermix column to remove 99% of the protein mass. Subsequent RP HPLC was performed to generate 20 fractions.

Although attractive for deep plasma analyses the Supermix column requires the higher volume of starting plasma, is not readily automated due the higher volumes and the characteristics of the separation are not considered defined enough for quantitative analysis. Nevertheless the increased protein identifications are clearly seen in Figure 3B compared to the automated 10 µL plasma shown in Figure 3A. Whilst starting with more material increases the number of proteins seen in the undepleted plasma, the use of immunodepletion significantly increases the coverage in both cases. In the automated 10 µL fractionation 169 proteins could be detected by a combination of undepleted and immunodepleted analysis with the subsequent fractionation in to 10 RP HPLCs increasing this to 321 proteins. With a non-automated fractionation of 250 µL this could be increased to 778 proteins with the extended immunodepletion adding 373 proteins not seen in undepleted analysis and the RP HPLC adding an additional 260 proteins.

## 4. Conclusions

The need for comprehensive plasma proteomics has been recognized for a long time [5,26]. The complexity and dynamic range of proteins requires prefractionation and several options have been reported [30]. Nanjappa *et al*., recently updated the data available for the human plasma proteome to include a collated list of 10,546 proteins of which 3784 were reported in at least two studies [31]. The process described here establishes and evaluates a comprehensive multi-dimensional workflow capable of high sensitivity. The important feature of the process described however is that by automation reproducible sample preparation is possible. This is potentially compatible with multiplexed label-free MS, targeted MS (e.g., selected reaction monitoring) or, alternatively, with any other protein detection methods.

We have compared three workflows, investigating different sensitivity levels: whole plasma, plasma immunodepleted to remove the abundant proteins and plasma which has been both immunodepleted and subsequently fractionated into either 10 or 20 fractions (10 µL plasma required). As expected, sensitivity was dramatically increased with fractionation. The mean CV% for all peptide peak intensities were 7.1% for repeated measurements (*i.e.*, reproducibility of LC-MS), 21.4% for the immunodepleted plasma and, perhaps most interestingly, 25.7% for the immunodepletion & fractionation workflow. As an example, an insulin-like growth factor protein (reported to be present in plasma at *circa* 15 ng/mL) was detected by five unique peptide sequences, from which ^74^SCDLALLETYCATPA^89^K peptide has the CV% ~ 11.7%. We provide three plasma protein catalogues obtained at different sensitivity levels as a browsable database of over 3000 peptide sequences (Table S1). In the current process a few limitations are notable. Firstly we were not able to assess biological variability within the scope of the study, however the aim was to increase sensitivity whilst retaining a high level of technical reproducibility biological variability is recognized as major factor and can be assessed in individual studies. Digestion is currently performed offline and further work will be needed to integrate this into the automated schema. Another challenge was that prefractionation inevitably leads to more sample processing. Despite these limitations, we feel that automation has been shown to be an important element in processing human plasma and has great potential in enabling clinical proteomics.

## Abbreviations

CSF: Cerebrospinal Fluid
MS: Mass Spectrometry
RP: Reversed Phase
SRM: Selected Reaction Monitoring

## Supplementary Files

Supplementary File 1 Supplementary Information (XLS, 20207 KB)
